# Thyroid Storm and Complete Heart Block after Treatment with Radioactive Iodine

**DOI:** 10.1155/2018/8214169

**Published:** 2018-06-11

**Authors:** Kaitlyn Vennard, Matthew P. Gilbert

**Affiliations:** Division of Endocrinology and Diabetes, The Robert Larner, M.D. College of Medicine, The University of Vermont, Burlington, VT, USA

## Abstract

Thyroid storm is a rare endocrine emergency characterized by dysfunction of multiple organ systems. Thyroid storm is more common in Graves' disease and can be precipitated by surgery, trauma, infection, metabolic abnormalities, iodine load, and parturition. We present a diagnostically challenging case of thyroid storm precipitated by radioiodine therapy and accompanied by bradycardia, a rare but life-threatening complication related to treatment for hyperthyroidism.

## 1. Introduction

Thyroid storm is a rare but potentially life-threatening condition induced by an excessive release of thyroid hormones. Most cases of thyroid storm occur after a precipitating event, intercurrent illness, or discontinuation of antithyroid medication. Radioiodine therapy for hyperthyroidism induces thyroiditis and subsequent fibrosis of the thyroid gland. The destruction of follicular cells within the thyroid gland results in the rapid release of stored thyroid hormone into the circulation, which can serve as a precipitating event for thyroid storm in a very small percentage of patients. However, it has proven difficult to define the risk factors for development of thyroid storm after radioiodine administration. Effective management of thyroid storm is predicated on the prompt recognition of the clinical signs and symptoms of this rare condition. However, there continues to be no consensus on the diagnostic criteria for thyroid storm. The most important determinates of survival in life-threatening thyroid storm are early recognition and institution of appropriate therapy. We present a case of thyroid storm precipitated by radioiodine therapy for hyperthyroidism and accompanied by bradycardia, a manifestation rarely observed in thyroid storm.

## 2. Case Description 

A 55-year-old woman with a history of type 2 diabetes mellitus, hyperlipidemia, obesity, and depression was referred to an endocrinologist with complaints of weight loss, palpitations, and diarrhea. The patient also had hypertension and was taking *α*-adrenergic receptor antagonists and a calcium channel blocker. She was found to have a thyroid-stimulating hormone (TSH) level of <0.10 (normal: 0.34 to 4.82) *µ*lU/ml and a free T4 concentration of 4.28 (normal: 0.6 to 1.6) ng/dL. I^123^ thyroid scan revealed elevated, diffuse uptake bilaterally, without nodules, consistent with the diagnosis of Graves' disease. The patient was treated with 11.9 mCi of radioactive iodine. Ten days after the ablation treatment, the patient presented to a local hospital by ambulance after experiencing lightheadedness, diffuse abdominal pain, and one episode of bilious emesis.

Upon arrival, she was hypotensive (77/44 mm Hg), pale, bradycardic, and febrile (39.4°C). An electrocardiogram (ECG) revealed accelerated junctional rhythm at a rate of 53 beats/min. The patient was given atropine 0.5 mg intravenously without effect, followed by initiation of external cardiac pacing. Continuous intravenous infusions of dopamine and norepinephrine were started along with fluid resuscitation of 4 L of normal saline over a 2-hour period. She received one ampule of calcium gluconate with no change in her blood pressure, heart rate, or rhythm. Computed tomography of the abdomen was unrevealing. The patient was endotracheally intubated and transferred via helicopter to our facility.

Upon arrival to our facility, she was receiving intravenous infusions of dopamine at 20 *µ*g/kg/min and norepinephrine at 10 *µ*g/kg/min and remained hypotensive (92/55 mm Hg) and bradycardic (59 beats/min). ECG showed an accelerated junctional rhythm. Laboratory findings included serum sodium of 139 mEq/L, potassium of 5.3 mEq/L, chloride of 108 mEq/L, and total CO_2_ content of 15 mmol/L, serum glucose of 208 mg/dL, urea nitrogen of 38 mg/dL, creatinine of 1.5 mg/dL, ionized calcium of 1.15 mmol/L, total bilirubin of 0.4 mg/dL, serum alkaline phosphatase of 141 U/L, aspartate aminotransferase of 2196 U/L, and alanine aminotransferase of 2010 U/L. Plasma troponin I was repeatedly undetectable. The peripheral leukocyte count was 15.9 x 10^9^ cells/L with no immature forms. There was evidence of an anion gap metabolic acidosis with a serum lactate concentration of 6.2 mmol/L. Blood cultures revealed no microbial growth. Thyroid function testing showed an undetectable TSH (<0.10 *µ*lU/ml), a free T4 of 12.8 (normal: 0.6 to 1.6) ng/dL, total T4 of 21.9 (normal: 5.6 to 13.7) *µ*g/dL, and a total T3 of 0.94 (normal: 0.8 to 1.8) ng/mL. The patient was treated for thyroid storm with 1000 mg of propylthiouracil by orogastric tube as a loading dose followed by 300 mg every 6 hours, 5 drops saturated solution of potassium iodide (SSKI) every 8 hours by orogastric tube, and 100 mg intravenously of hydrocortisone every 8 hours. The hypotension resolved, vasopressors were stopped, and the patient was extubated 25 hours after her initial presentation. During her stay in the intensive care unit, the patient exhibited fever (39.6°C maximum), tachycardia, and tremulousness. These manifestations resolved over a period of 22 hours. The patient was discharged in satisfactory condition on the fourth day of hospitalization on 100 mg of propylthiouracil orally every 8 hours. Outpatient testing days later demonstrated normalization of her thyroid function tests.

## 3. Discussion 

Radioiodine treatment for hyperthyroidism secondary to Graves' disease has been shown to be safe and effective and remains the treatment of choice in North America. Although hypothyroidism remains the most common complication of radioiodine therapy, more severe complications can occur. The incidence of thyroid storm among patients with hyperthyroidism is estimated at 1% to 2% [1]. The mortality rate in thyroid storm has been reported as high as 20% to 50%, even with appropriate treatment [2].

Thyrotoxicosis and thyroid storm have been previously reported after radioiodine therapy [3, 4]. A comprehensive review of cases of thyroid storm after radioiodine therapy estimated the frequency to be 0.34% [4]. Radioiodine therapy induces thyroiditis and subsequent fibrosis of the thyroid gland. The destruction of follicular cells within the thyroid gland results in the release of stored thyroid hormone into the circulation. It has proven difficult to define the group of patients at risk for the development of thyroid storm after radioiodine administration [4]. Methods to effectively decrease the risk of complications after radioiodine therapy, such as pretreatment with antithyroid drugs, remain controversial [5].

Hyperthyroidism is commonly associated with a spectrum of cardiovascular abnormalities. Rhythm disturbances, particularly atrial arrhythmias and sinus tachycardia, are frequently encountered in patients with hyperthyroidism [6]. Disturbances of atrioventricular (AV) conduction are less common but have been reported in patients with thyrotoxicosis and thyroid storm [7–9]. Intra-atrial and intraventricular conduction disturbances each occur in approximately 15% of patients with thyrotoxicosis [6]. The majority of cases of complete heart block associated with thyrotoxicosis and thyroid storm have been associated with additional risk factors for conduction abnormalities. These factors include infection [7], coexisting cardiac anomalies [8], digoxin use [10], and electrolyte abnormalities including hypercalcemia [11] and hypokalemia [12]. However, in some cases, no underlying disease was identified and subsequent treatment of the thyrotoxicosis resulted in restoration of normal AV conduction [8, 9].

We present a case of thyroid storm precipitated by radioiodine therapy and manifesting as heart block, both rare but life-threatening complications of treatment for hyperthyroidism. The classic presentation of thyroid storm includes fever, tachycardia, tremor, nausea and vomiting, diarrhea, dehydration, delirium, and coma [13]. The lack of tachycardia can be explained by her complete heart block. The patient did develop tachycardia later in the hospital after resolution of her complete heart block. Interestingly, our patient had none of the previously reported risk factors for development of complete heart block associated with thyrotoxicosis and thyroid storm. An ECG revealed no coexisting cardiac anomalies; she was not hypokalemic nor hypercalcemic and was not taking digoxin. The patient's rapidly developing symptoms, clinical signs, and laboratory data coupled with her rapid recovery following initiation of antithyroid treatment support the diagnosis of thyroid storm likely precipitated by radioactive iodine treatment.

The patient also had evidence of multiorgan system failure including acute renal and hepatic dysfunction, hypotension, and lactic acidosis. Several factors contributed to the lactic acidosis in our patient. The complete heart block resulted in a decrease in cardiac output and subsequent hypotension and led to tissue hypoperfusion and increased lactate production. In addition, our patient's hepatic dysfunction reduced the clearance of lactate from the bloodstream. Blood and urine cultures revealed no bacterial growth and sepsis was ruled out as an etiology of our patient's multiorgan system failure. While mild elevations of liver enzymes are common in hyperthyroidism, hepatic failure is rare [14]. Jiang et al. (2000) noted severe hepatic dysfunction in their report of thyroid storm presenting as multiorgan system failure [15].

The development of thyroid storm following ablative treatment for hyperthyroidism and the occurrence of bradycardia in the setting of thyroid storm both represent uncommon manifestations of treatment for hyperthyroidism. Timely recognition of this atypical presentation of thyroid storm allowed prompt implementation of potentially life-saving treatment.
